# ^1^H, ^15^N and ^13^C resonance assignments for free and IEEVD peptide-bound forms of the tetratricopeptide repeat domain from the human E3 ubiquitin ligase CHIP

**DOI:** 10.1007/s12104-016-9710-y

**Published:** 2016-10-05

**Authors:** Huaqun Zhang, Cameron McGlone, Matthew M. Mannion, Richard C. Page

**Affiliations:** Department of Chemistry and Biochemistry, Miami University, 651 E. High St, Oxford, OH 45056 USA

**Keywords:** CHIP, TPR, Ubiquitin ligase, Heat shock proteins, NMR

## Abstract

The ubiquitin ligase CHIP catalyzes covalent attachment of ubiquitin to unfolded proteins chaperoned by the heat shock proteins Hsp70/Hsc70 and Hsp90. CHIP interacts with Hsp70/Hsc70 and Hsp90 by binding of a C-terminal IEEVD motif found in Hsp70/Hsc70 and Hsp90 to the tetratricopeptide repeat (TPR) domain of CHIP. Although recruitment of heat shock proteins to CHIP via interaction with the CHIP-TPR domain is well established, alterations in structure and dynamics of CHIP upon binding are not well understood. In particular, the absence of a structure for CHIP-TPR in the free form presents a significant limitation upon studies seeking to rationally design inhibitors that may disrupt interactions between CHIP and heat shock proteins. Here we report the ^1^H, ^13^C, and ^15^N backbone and side chain chemical shift assignments for CHIP-TPR in the free form, and backbone chemical shift assignments for CHIP-TPR in the IEEVD-bound form. The NMR resonance assignments will enable further studies examining the roles of dynamics and structure in regulating interactions between CHIP and the heat shock proteins Hsp70/Hsc70 and Hsp90.

## Biological context

The C-terminus of Hsc70 interacting protein (CHIP) is a ubiquitin ligase involved in protein degradation by the ubiquitin–proteasome pathway, as well as a co-chaperone of the 70- and 90-kDa heat shock proteins (Hsp70/Hsc70 and Hsp90). CHIP is comprised of three domains, a tetratricopeptide repeat (TPR) domain (D’Andrea and Regan 2003) at the N terminus, a C-terminal U-box domain, and a helical linker domain located between the TPR and U-box (Zhang et al. 2005). CHIP binds to the C-terminal IEEVD motif of heat shock proteins through the TPR domain (Zhang et al. 2005, 2015), while the U-box domain is required for E3 ligase activity (Connell et al. 2001). Multiple studies have identified the CHIP-TPR domain as necessary for binding to heat shock proteins or other proteins that harbor a C-terminal IEEVD motif (D’Andrea and Regan 2003; Zhang M et al. 2005; Zhang H et al. 2015).

CHIP was first discovered as a co-chaperone and negative regulator of chaperone functions (Ballinger et al. 1999). Further investigation confirmed that CHIP acted as a E3 ubiquitin ligase (Connell et al. 2001). Previous research also found that CHIP preferentially participated in ubiquitination of Hsp70-bound clients, thus affecting protein triage decisions (Stankiewicz et al. 2010). CHIP plays an essential role in many physiological processes including DNA repair, processing of antigens by the immune system, neurological disorders, cardiac diseases, muscular disorders, and cancers (Paul and Ghosh 2015). Important targets of CHIP related to cancers include, but are not limited to ErbB2, TRAF2, PTEN, p53, and AKT (Paul and Ghosh 2015). CHIP up-regulation can inhibit tumor growth and metastasis and its level was inversely correlated with the malignancy of human breast and gastric tumors (Paul and Ghosh 2015) as well as pancreatic tumors. Given its diverse roles, CHIP is a clinically important protein with significant potential to serve as a therapeutic target.

**Fig. 1:**
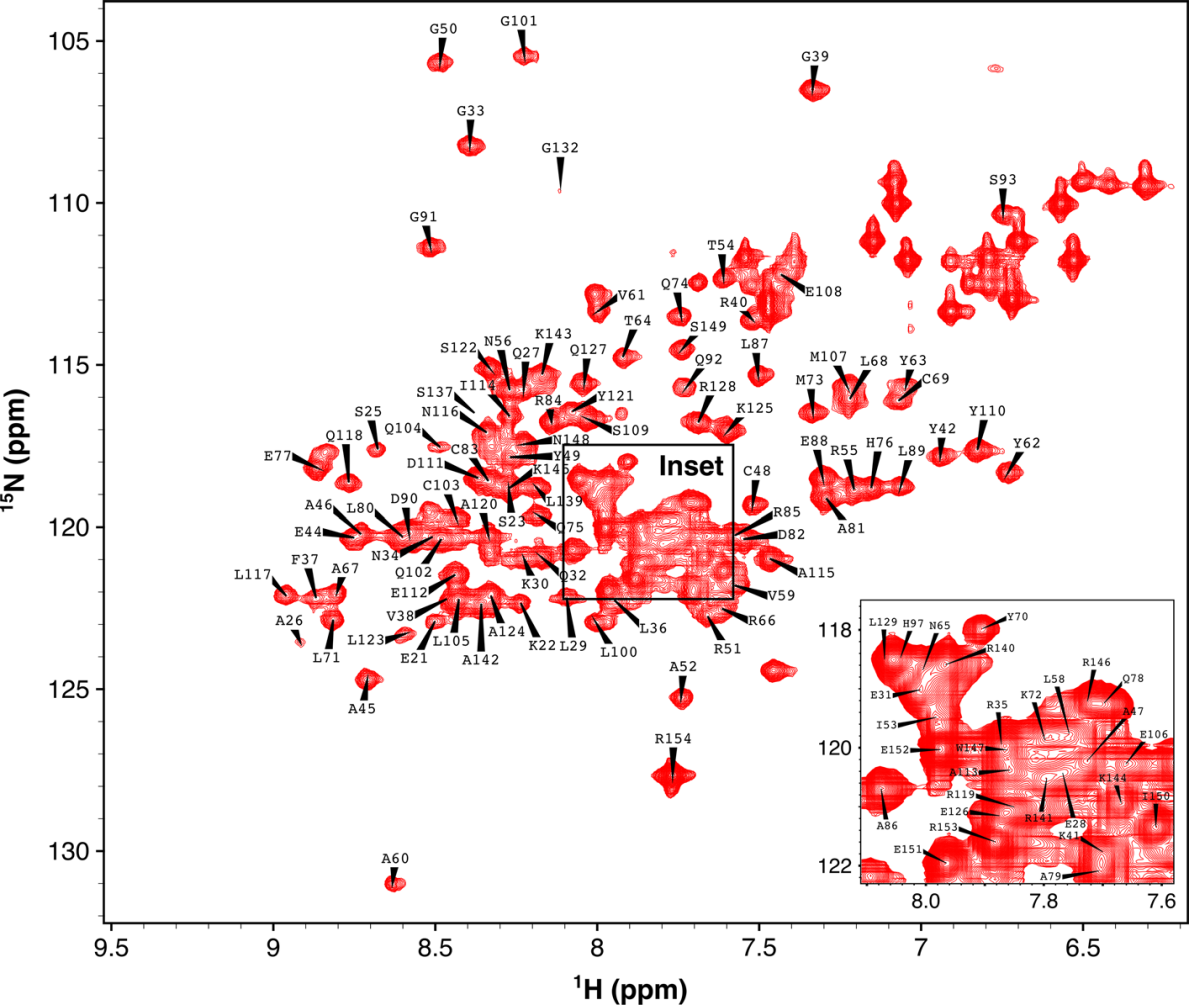
^1^H/^15^N-HSQC of CHIP-TPR in the free form. *Inset* expands the crowded region of the spectrum. Backbone amide resonances are labeled according to amino acid residue type and number. A complete list of resonance assignments can be found in the BMRB repository under accession no. 26818

**Fig. 2:**
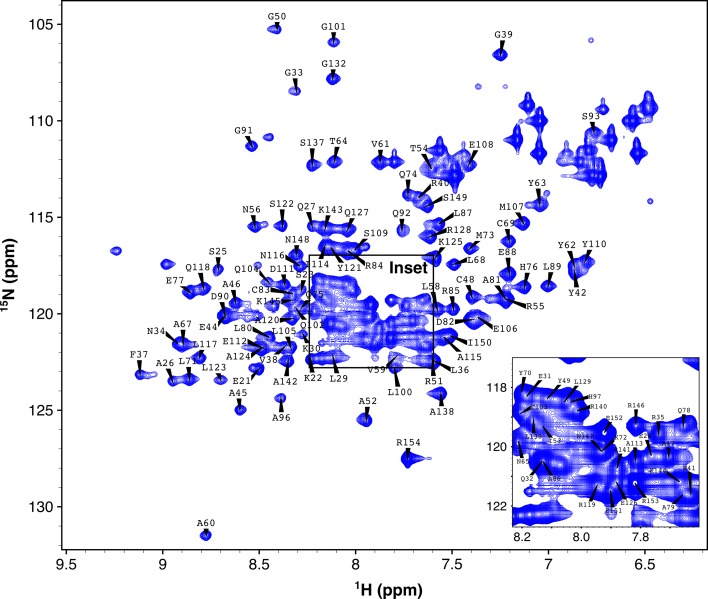
^1^H/^15^N-HSQC of CHIP-TPR in complex with the Hsp70-IEEVD peptide. *Inset* expands the crowded region of the spectrum. Backbone amide resonances are labeled according to amino acid residue type and number. A complete list of resonance assignments can be found in the BMRB repository under accession no. 26819

TPR domains are a structural motif typically consisting of between three to sixteen repeats of a 34-amino acid sequence that produces a helix-turn-helix structure (D’Andrea and Regan 2003). The CHIP-TPR is comprised of three TPR repeats followed by a seventh helix which leads to the CHIP linker domain. TPR domains exist in a diverse range of proteins and organisms, and are believed to function as protein–protein interaction domains (D’Andrea and Regan 2003). Within the context of CHIP, the TPR domain interacts with Hsp70/Hsc70 and Hsp90 through binding to a C-terminal IEEVD motif. Although crystal structures of CHIP-TPR in complex with IEEVD motifs are known (Zhang M et al. 2005; Zhang H et al. 2015), the structure in the free form is not known. A recent hydrogen/deuterium exchange mass spectrometry (HDX-MS) study found that the TPR domain of CHIP was significantly more dynamic in the free form, whereas it was stabilized upon binding to full-length heat shock proteins or C-terminal IEEVD motif peptides (Graf et al. 2010). The HDX-MS study suggests that the TPR domain exhibits a substantial conformational changes or decreases in dynamics after binding to IEEVD motifs. For therapeutic targeting of CHIP aimed at disrupting interactions with heat shock proteins the structure and dynamics of free CHIP-TPR represent valuable information that could inform rational drug design. NMR is uniquely suited for examining both dynamics and structure and both backbone and side chain chemical shift assignments of CHIP-TPR are important first steps toward structure determination and studies of dynamics for free-form CHIP-TPR using solution NMR. Here we report the backbone and side-chain resonance assignments for CHIP-TPR in its free form (Fig. 1), and backbone resonance assignments for CHIP-TPR in complex with an Hsp70-tail IEEVD motif (Fig. 2).

**Fig. 3:**
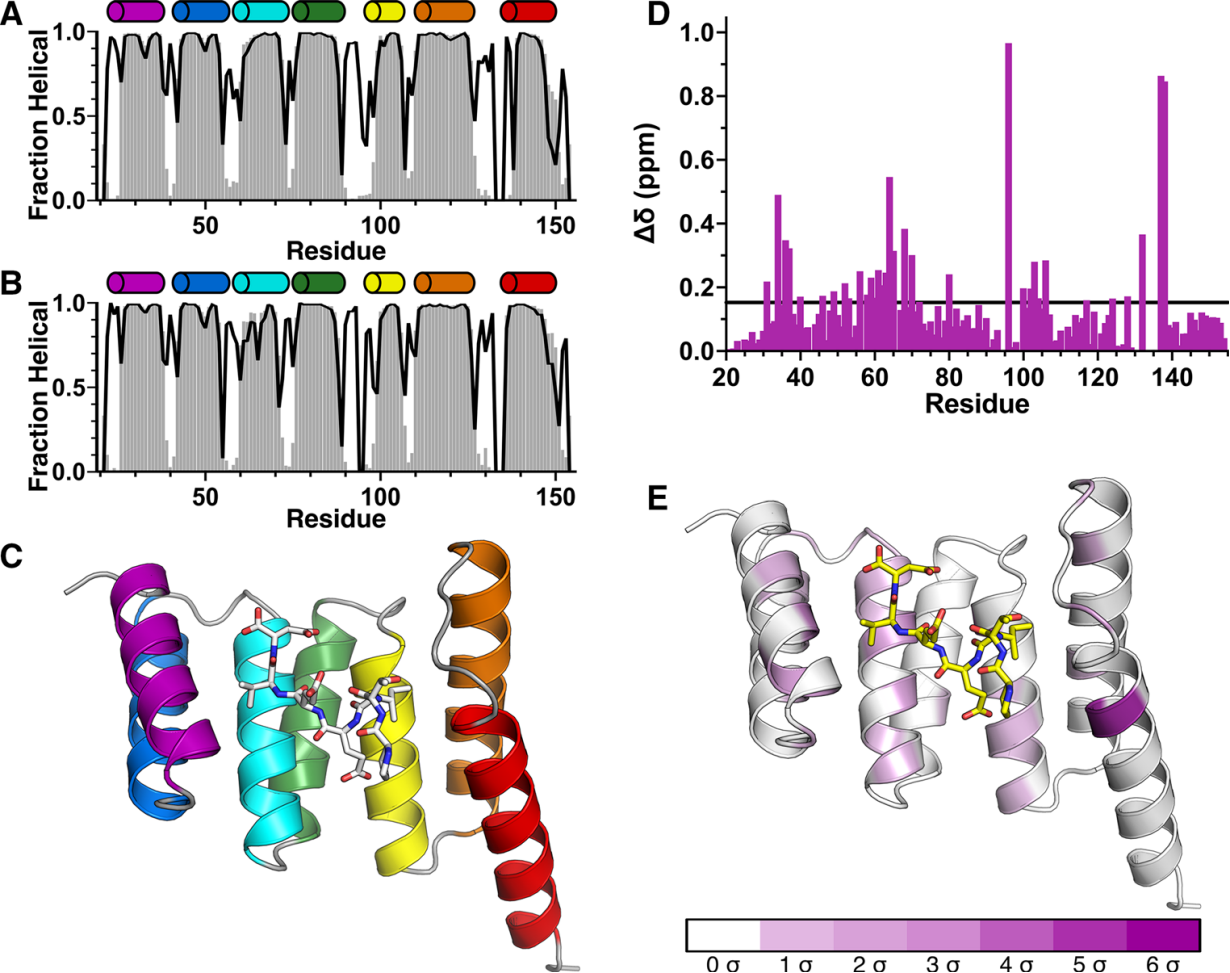
Secondary structure predicted by CHIP-TPR resonance assignments. Fraction helical content predicted by TALOS+ (Shen et al. 2009) for CHIP-TPR in the free form (**a**) and in complex with the Hsp70-IEEVD peptide (**b**). The *solid black line* indicates the per residue prediction confidence, on a *scale* of 0–1.0, reported by TALOS+. Above each *plot cylinders* representing TPR helices 1–7 are shown *colored* according to position within the structure of CHIP-TPR (**c**) in complex with the Hsp70-IEEVD peptide (*white sticks*) as observed in the crystal structure of the CHIP-TPR/Hsp70 complex from PDB accession ID 4 kbq (Zhang et al. 2015). Chemical shift perturbations (δΔ) from ^1^H/^15^N-HSQC spectra that occur upon binding of the Hsp70-IEEVD to CHIP-TPR (**d**) are shown as *purple bars*. A *black horizontal line* indicates one standard deviation (σ). Chemical shift perturbations greater than one σ are mapped on to the crystal structure (**e**) of the CHIP-TPR/Hsp70-IEEVD complex from PDB accession ID 4kbq (Zhang et al. 2015). Chemical shift perturbations are colored according to the *scale* shown (**e**) and the Hsp70-IEEVD is shown as *yellow sticks*

Backbone resonance assignments in the free form (Fig. 1) and the IEEVD-bound form (Fig. 2) are consistent with the X-ray crystal structure of the CHIP-TPR/Hsp70-IEEVD complex (Fig. 3), as indicated by secondary structure predictions from TALOS+ (Shen et al. 2009). Of the 116 assigned backbone NH pairs, Ala60 stands out as it exhibits a significant downfield shift, possibly due to ring current effects from the phenyl rings of Tyr62 and Tyr63, and the positioning of Ala60 at the N-terminus of helix 3. While the TALOS+ secondary structure predictions do not suggest significant differences between free and IEEVD-bound forms of CHIP-TPR, chemical shift perturbations (Fig. 3d, e) tell a slightly different story. Mapping chemical shift perturbations that arise from binding of the Hsp70-IEEVD to CHIP-TPR (Fig. 3e) onto the CHIP-TPR structure identifies residues that lie at the interaction surface and residues that do not appear to directly contact the IEEVD. Those residues that do not appear to directly contact the IEEVD instead mediate contacts between CHIP-TPR helices. This behavior is consistent with a recent HDX-MS study of CHIP (Graf et al. 2010) which found that the CHIP-TPR exhibits higher dynamics than the CHIP linker or U-box domains, and dynamics within the CHIP-TPR decrease significantly upon binding of the Hsp70-IEEVD. Interpreted in light of previous HDX-MS data, chemical shift perturbations between CHIP-TPR helices suggest that while secondary structure content remains unchanged, the relative positions of helices likely change between the free and IEEVD-bound states. Anecdotally, the chemical shift perturbations and previous HDX-MS data suggest that the absence of a CHIP-TPR crystal structure in the free form may not be for lack of effort, but rather because conformational variability or dynamics of free form CHIP-TPR preclude the formation of diffraction quality crystals. This dynamic behavior of CHIP-TPR suggests that solution NMR studies, enabled by the resonance assignments reported here, will be important for gaining structural insights for CHIP-TPR in the absence of an IEEVD peptide.

## Methods and experiments

### Protein expression and purification

The gene encoding CHIP-TPR(21-154) was cloned into the pHis||2 expresssion vector as previously reported (Zhang et al. 2015), enabling expression of a construct with a His_6_ tag at the N-terminus followed by a TEV protease cleavage site. The recombinant plasmid was transformed into R2D competent cells, which were then spread onto LB plates containing 100 µg/mL ampicillin. After incubating at 37 °C overnight, colonies were picked to inoculate 20 mL LB start culture with 100 µg/mL ampicillin. Cells were grown overnight with shaking at 37 °C and used the next morning for inoculating a 500 mL buffer-enhanced minimal media culture. For ^15^N or ^13^C/^15^N labeled protein, cells from the overnight culture were centrifuged, LB media was decanted and the cell pellet was resuspended in minimal media supplemented with 2 g/L ^15^NH_4_Cl and 2 g/L glucose (^13^C). Inoculated cultures, typically 500 mL in volume, were grown to an OD_600_ between 0.6 and 0.8, then transferred to 17 °C shaker for 1 h followed by addition of 0.4 mM IPTG. Induced cultures were grown at 17 °C overnight, cells were harvested by centrifugation and resuspended in lysis buffer (180 mM Tris, 450 mM NaCl, 10 % glycerol, 5 mM β-mercaptoethanol, pH7.8). Harvested cells were frozen in liquid nitrogen and stored at −80 °C until purification.

Harvested cells were thawed overnight with rotation at 4 °C followed by sonication, after which lysate was pelleted by centrifugation at 11,500 rpm for 45 min at 4 °C. The supernatant was filtered with a 0.4 µm syringe filter and transferred to a new 50 mL centrifuge tube, and after filtration supernatant was loaded onto a HisTrap column (GE Healthcare) for affinity chromatography purification. The HisTrap column was washed by buffer A (20 mM Hepes, 150 mM NaCl, 5 mM β-mercaptoethanol, pH7.0) followed by wash by 5 % and elution by 100 % buffer B (20 mM Hepes, 150 mM NaCl, 500 mM imidazole, 5 mM β-mercaptoethanol, pH7.0). Eluted protein was subjected to TEV protease cleavage by dialysis against cleavage buffer (20 mM Hepes, 50 mM NaCl, 1 mM EDTA, 5 mM β-mercaptoethanol, pH7.5) at 4 °C overnight. Cleaved protein was concentrated to 1 mL and loaded onto a Superdex 75 column (GE Healthcare) for further purification. Fractions containing CHIP-TPR were concentrated to approximately 0.5 mM for 2D or 3D NMR data acquisition.

### Nuclear magnetic resonance (NMR) spectroscopy

To assign the chemical shifts of CHIP-TPR backbone atoms ^1^H/^15^N-HSQC, ^1^H/^15^N/^13^C-HNCO, CBCA(CO)NH and HNCACB experiments were carried out on a Bruker 600 MHz spectrometer equipped with a triple resonance ^1^H/^13^C/^15^N probe. For side chain chemical shift assignments of CHIP-TPR, HCCH-TOCSY and CCH-COSY experiments together with 2D ^13^C-HSQC were performed on a Bruker 600 MHz spectrometer. All experiments were performed at 293 K with CHIP-TPR concentration of 0.5 mM in buffer containing 10 % D_2_O. All spectra for backbone and side-chain chemical shifts of CHIP-TPR were processed using NMRPipe (Delaglio et al. 1995) and analyzed using Sparky.

## Assignment and data deposition

Backbone amide assignments of CHIP-TPR are shown overlaid onto ^15^N-HSQC spectra in the free form (Fig. 1) and the IEEVD-bound form (Fig. 2). 116 backbone amides out of 134 were assigned in total. In our data, the region from N130 to S137 cannot be assigned due to very weak signals, possibly caused by high flexibility. This same region was identified as highly flexible by an earlier HDX MS study (Graf et al. 2010). For assigned backbone amides, nearly all corresponding side chain chemical shifts were assigned. Backbone and side chain chemical shift assignments of free CHIP- TPR have been deposited into BioMagResBank (http://www.bmrb.wisc.edu) with accession no. 26818. Backbone chemical shift assignments for IEEVD-bound CHIP-TPR have been deposited into BioMagResBank (http://www.bmrb.wisc.edu) with accession no. 26819. Based on backbone chemical shifts for H_α_, C_α_, C_β_, C_O_, H_N_, and N_H_ atoms, secondary structures were predicted for CHIP-TPR in both free and IEEVD-bound forms out using TALOS+ (Shen et al. 2009). The TALOS+ predictions (Fig. 3) identify seven alpha helices, consistent with the crystal structure of CHIP-TPR in complex with Hsp70 peptide (Fig. 3c). Although minor changes in helical content were observed, the most significant changes occurred in helices 3 and 7 which each contain multiple residues that interact with the bound IEEVD motif. Chemical shift perturbations upon binding of Hsp70-IEEVD to the CHIP-TPR were calculated as shown in Eq. 1.

1 $$\Delta \delta = \sqrt {\left( {\Delta {}_{{}}^{1} H_{N} } \right)^{2} + \left( {{\raise0.7ex\hbox{${\Delta {}_{{}}^{15} N_{H} }$} \!\mathord{\left/ {\vphantom {{\Delta {}_{{}}^{15} N_{H} } 5}}\right.\kern-0pt} \!\lower0.7ex\hbox{$5$}}} \right)^{2} }$$
